# Identification of a seven-lncRNAs panel that serves as a prognosis predictor and contributes to the malignant progression of laryngeal squamous cell carcinoma

**DOI:** 10.3389/fonc.2023.1106249

**Published:** 2023-05-02

**Authors:** Xiwang Zheng, Wei Gao, Zhe Zhang, Xuting Xue, Maierhaba Mijiti, Qingbo Guo, Dilinaer Wusiman, Kai Wang, Xianhai Zeng, Lingbin Xue, Zekun Guo, Changming An, Yongyan Wu

**Affiliations:** ^1^ Shanxi Key Laboratory of Otorhinolaryngology Head and Neck Cancer, First Hospital of Shanxi Medical University, Taiyuan, Shanxi, China; ^2^ Shanxi Province Clinical Medical Research Center for Precision Medicine of Head and Neck Cancer, First Hospital of Shanxi Medical University, Taiyuan, Shanxi, China; ^3^ Department of Otolaryngology Head & Neck Surgery, Longgang Otolaryngology Hospital, Shenzhen, Guangdong, China; ^4^ Shenzhen Institute of Otolaryngology & Key Laboratory of Otolaryngology, Longgang Otolaryngology Hospital, Shenzhen, Guangdong, China; ^5^ Shenzhen Eye Hospital, Jinan University, Shenzhen Eye Institute, Shenzhen, Guangdong, China; ^6^ Department of Head and Neck Surgery, National Cancer Center/National Clinical Research Center for Cancer/Cancer Hospital, Chinese Academy of Medical Sciences and Peking Union Medical College, Beijing, China; ^7^ Department of Otolaryngology Head & Neck Surgery, Southern University of Science and Technology Hospital, Southern University of Science and Technology, Shenzhen, Guangdong, China; ^8^ College of Life Sciences, Northwest A&F University, Yangling, Shaanxi, China

**Keywords:** laryngeal squamous cell carcinoma, biomarker, long non-coding RNA, oncogene, prognosis

## Abstract

**Background:**

Laryngeal squamous cell carcinoma (LSCC) is one of the most frequent head and neck cancers worldwide. Long non-coding RNAs (lncRNAs) play a critical role in tumorigenesis. However, the clinical significance of lncRNAs in LSCC remains largely unknown.

**Methods:**

In this study, transcriptome sequencing was performed on 107 LSCC and paired adjacent normal mucosa (ANM) tissues. Furthermore, RNA expression and clinical data of 111 LSCC samples were obtained from The Cancer Genome Atlas (TCGA) database. Bioinformatics analysis were performed to construct a model for predicting the overall survival (OS) of LSCC patients. Moreover, we investigated the roles of lncRNAs in LSCC cells through loss-of-function experiments.

**Results:**

A seven-lncRNAs panel including ENSG00000233397, BARX1-DT, LSAMP-AS1, HOXB-AS4, MNX1-AS1, LINC01385, and LINC02893 was identified. The Kaplan–Meier analysis demonstrated that the seven-lncRNAs panel was significantly associated with OS (HR:6.21 [3.27-11.81], p-value<0.0001), disease-specific survival (DSS) (HR:4.34 [1.83-10.26], p-value=0.0008), and progression-free interval (PFI) (HR:3.78 [1.92-7.43], p-value=0.0001). ROC curves showed the seven-lncRNAs panel predicts OS with good specificity and sensitivity. Separately silencing the seven lncRNAs inhibited the proliferation, migration, and invasion capacity of LSCC cells.

**Conclusion:**

Collectively, this seven-lncRNAs panel is a promising signature for predicting the prognosis of LSCC patients, and these lncRNAs could serve as potential targets for LSCC treatment.

## Introduction

Laryngeal squamous cell carcinoma (LSCC) is the second most frequent malignancy of the head and neck, or the upper respiratory tract (1, 2). Despite the improvements in curative techniques, laryngeal cancer is still one of the few cancers in which the 5-year survival rate continues to decline, over the past 40 years (3, 4). LSCC has the malignant characteristics of local invasion and cervical lymph node metastasis, which are major risk factors of mortality for LSCC patients (5, 6). Consequently, about 60% of LSCC patients have already progressed to the advanced clinical stages (III/IV) at diagnosis, and thus missed the optimal therapeutic window (7).

Molecular biomarkers or signatures are extremely valuable tools for the early diagnosis and accurate prognosis of cancer in modern times (8, 9). Long non-coding RNAs (lncRNAs) are a class of non-protein coding RNA molecules with more than 200 nucleotides (10) that have a variety of regulatory functions on cancer cell proliferation, genomic stability, metabolism, angiogenesis, immunity, and metastasis (11–16). Moreover, lncRNAs were considered important candidate biomarkers due to their specific spatiotemporal expression and diversity in cancer (17). Multiple studies reported that a variety of lncRNAs were identified as promising biomarkers for the diagnosis and prognosis of cancers (18–20), and some of these are already being used in clinical trials (21, 22). Furthermore, it has been shown that lncRNAs are involved in regulating the progression of LSCC, and may therefore be used as potential disease biomarkers (23, 24). Unfortunately, there are currently no molecular biomarkers or signatures used for the diagnosis and prognosis of LSCC. Hence, it is necessary to explore more putative lncRNA biomarkers or signatures with high specificity and sensitivity for LSCC.

High-throughput RNA sequencing (RNA-Seq) provides an efficient approach for the identification of differentially expressed genes in large-scale samples. In this study, RNA-Seq was performed to screen for differentially expressed lncRNAs in 107 LSCC and paired adjacent normal mucosa (ANM) tissues (Shanxi cohort). Furthermore, the lncRNAs expression and clinical data of 111 LSCC and 12 normal samples were obtained from The Cancer Genome Atlas (TCGA) database. By integrating the Shanxi cohort and the TCGA cohort data, we investigated the lncRNA expression profiles, determined the pathways involved with dysregulated lncRNAs, and identified lncRNAs associated with the survival of LSCC patients. We constructed a seven-lncRNAs signature with high specificity and sensitivity and developed a nomogram for predicting the prognosis of LSCC patients. Lastly, we explored the functional roles of the seven lncRNAs in LSCC cells. Our findings provide novel potential biomarkers and molecular targets for the prognosis evaluation and therapy of LSCC.

## Materials and methods

### Tissue samples

LSCC tissues and paired ANM tissues were collected from patients who had undergone surgery at the Department of Otolaryngology Head and Neck Surgery, First Hospital of Shanxi Medical University. None of the patients received chemotherapy or radiotherapy before surgery. The clinical samples were collected from LSCC patients after obtaining their informed consent. Studies were performed with the approval of the medical ethics committee of First Hospital of Shanxi Medical University.

### RNA isolation, library preparation, and RNA-sequencing

Total RNA was isolated using TRIzol reagent (Ambion, USA) following the manufacturer’s protocol. After evaluation of purity, concentration, and integrity, 3 μg of RNA per sample was used in library preparation for RNA sequencing. The rRNA was removed using the Ribo-zero rRNA Removal Kit (Epicentre, USA), and the rRNA-depleted sample was subsequently used to generate sequencing libraries using the NEB Next Ultra Directional RNA Library Prep Kit for Illumina (NEB, USA) according to the manufacturer’s protocols. In brief, the rRNA-depleted RNA samples were subjected to RNA fragmentation, first strand cDNA synthesis with random hexamer primers, second strand cDNA synthesis with dUTP, adenylation of 3’ ends, barcoded adapter ligation, purification, and amplification using PCR to generate sequencing libraries. After cluster generation, libraries were sequenced on an Illumina HiSeq 4000 platform by Novogene (Beijing, China).

### Analysis of RNA-sequencing data

Clean reads were obtained by removing reads adapter, reads containing poly-N, and low-quality reads from raw data. The reads mapping and transcript assembly was done using TopHat (v2.0.9) and Cufflinks (v2.1.1) based on the human reference genome (GRCh37) from the Ensembl database (https://www.ensembl.org/). Then, the coding potential of transcripts was predicted using CPC2 (beta) and CNCI (v2). Finally, the read counts in fragments per kilobase million (FPKM) of transcripts were used as input data for a more in-depth bioinformatic analysis. The TCGA expression data of LSCC were obtained from the TCGA data portal (https://cancergenome.nih.gov/). The associated clinical features were obtained from the University of California, Santa Cruz Xena (UCSC Xena; https://xenabrowser.net/) database. The significantly differentially expressed lncRNAs in the Shanxi and TCGA cohorts were screened using the R package DEseq2 (v1.26.0) (25) with the cut-off p-value< 0.05 and | log_2_(Fold change) | ≥ 1.

### Unsupervised learning analysis

Complete-linkage clustering, which is one of the hierarchical clustering algorithms, was used to analyze the differential expression of lncRNAs in relation to various clinical features. The heat map to visualize the expression data was constructed using the R package pheatmap (v1.0.12, https://CRAN.R-project.org/package=pheatmap). Principal component analysis (PCA) was used to reduce the dimensionality of high dimensional expression datasets and evaluate the degree of difference between LSCC and normal tissue. The 3D visualization of PCA was realized using the R package pca3d (v0.10.1, https://CRAN.R-project.org/package=pca3d).

### Gene set enrichment analysis

Functional annotation of dysregulated lncRNAs in LSCC was performed using the GSEA (v3.0) software with Molecular Signatures Database (v7.0, https://www.gsea-msigdb.org/gsea/msigdb/). Significantly upregulated and downregulated lncRNAs in both the Shanxi and TCGA cohorts were obtained by Venn analysis. Pearson correlation analysis with Student’s *t*-test was used to obtain protein-coding RNAs that are correlated with lncRNAs based on their expression levels, with a cut-off set at | Pearson’s *r* | ≥0.4 and p-value< 0.05. Based on the ranking of Pearson’s *r*, the five most positively and five most negatively correlated protein-coding RNAs to each of the lncRNAs were selected as the input data for GSEA. Mutual functional pathways in two LSCC cohorts were mined using a cut-off set at FDR q-value ≤ 0.01 after GSEA.

### Prediction of the biological processes and pathways associated with the identified seven lncRNAs

The previously identified protein-coding RNAs that were correlated with the expression of the seven lncRNAs were used to determine the different associated biological signaling pathways using Metascape (https://metascape.org/).

### Survival analysis

Univariate Cox proportional hazards regression analysis was used to investigate the relationship between the continuous expression levels of each significantly dysregulated lncRNA and the overall survival (OS) of patients in the TCGA cohort. A total of 111 LSCC patients’ data from TCGA database were used for survival analysis, in which 50 patients were reached the survival endpoint. Based on the expression fold-change of each lncRNA, the output of the Cox regression analysis was divided into an upregulation set and a downregulation set. Under the criterion of p-value< 0.05, 20 lncRNAs in the upregulation set and 4 lncRNAs in the downregulation set were defined as significant high-risk lncRNAs for LSCC patients. The least absolute shrinkage and selection operator (LASSO) algorithm with a penalty parameter of 1000 and a 10-fold cross-validation was used to shrink sparse high-dimensional data and develop a prognostic prediction model from the output of the Cox regression analysis. The LASSO algorithm was implemented using the R package glmnet (v2.0_16, https://CRAN.R-project.org/package=glmnet). To determine the risk levels of the prognostic prediction model for OS, the risk score for each LSCC patient was calculated using the formula: $\text{risk score = }\overset{\text{n}}{\underset{\text{i}=1}{\sum }}{(\text{c}}_{\text{lncRNAi}}{\;\times \text{ e}}_{\text{lncRNAi}})$ ; where $c_{lncRNAi}$ is the relation coefficient of the risk factor *lncRNAi*; ${\text{e}}_{lncRNAi}$ is the expressional value of the risk factor *lncRNAi*; and *n* is the number of lncRNAs included in the prognostic prediction model. The LSCC patients were classified into high or low risk groups according to the median value of the computed risk scores. The Kaplan-Meier (K-M) estimator model with the log-rank test was used to estimate the OS, disease-specific survival (DSS), and progression-free interval (PFI) for LSCC patients between the high and low risk groups. The survival curve was realized using the R packages survival (v2.44-1.1, https://cran.r-project.org/package=survival) and survminer (v0.4.4, https://CRAN.R-project.org/package=survminer). The sensitivity and specificity of the lncRNAs prognostic prediction model were analyzed using a time-dependent receiver operating characteristic (ROC) curve. The area under the curve (AUC) was compared among the 1-, 3- and 5-year survival rates. The calculation and visualization of the ROC curves were done using the R package timeROC (v0.3, https://CRAN.R-project.org/package=timeROC). Moreover, a total of 86 LSCC samples with follow-up data was used to validate the efficiency of prognostic model. The expression levels of 7 lncRNAs in the 86 LSCC samples were determined by qPCR analysis. A nomogram for predicting OS was built using the R package rms (v5.1-3, https://CRAN.R-project.org/package=rms). The survival probabilities were predicted for the 1-, 3- and 5-year marks. The validation of the nomogram prediction model was accessed using bootstrapped calibration curves.

### Construction of the lncRNAs-miRNAs-mRNAs network

The correlation among seven lncRNAs was analyzed using the R package stats (v4.1.0) and visualized using the R package corrplot (v 0.92, https://cran.r-project.org/package=corrplot). An lncRNAs-miRNAs-mRNAs network was constructed based on competitive endogenous RNA (ceRNAs). For target miRNA prediction of seven lncRNAs, miRanda (v3.3a, https://anaconda.org/bioconda/miranda), LncTar (v1.0, http://www.cuilab.cn/lnctar) and RNA22 (v2.0, https://cm.jefferson.edu/rna22/) were used to independently predict the binding between lncRNAs and miRNAs. Next, the top 30 miRNAs with strongest binding ability to each lncRNA in three softwares were intersected to obtained potential target miRNAs. For target mRNA prediction of miRNAs, Targetscan (v7.1, https://www.targetscan.org/), miRdb (https://mirdb.org/mirdb/) and mirWalk (http://mirwalk.umm.uni-heidelberg.de/) were utilized. Target Score >=20 in miRDB, energy<= -5 in miRWalk and Total context++ score<= -0.1 in Targetscan were used as the threshold, and target mRNAs for each miRNA were obtained from intersection of three databases. Finally, Cytoscape (v3.7.1. https://cytoscape.org/) was used to construct the lncRNAs-miRNAs-mRNAs network.

### Cell culture, transfection, and quantitative real-time PCR

Human LSCC TU-177 cell lines (Bioleaf Biotech, China) were cultured in Dulbecco’s Modified Eagle’s Medium (DMEM; Life Technologies, USA) supplemented with 10% fetal bovine serum (FBS; BI, USA). Human LSCC FD-LSC-1 (26) cell lines were cultured with Bronchial Epithelial Cell Growth Medium (BEGM; Lonza, Switzerland) supplemented with 10% FBS. All cells were maintained in a humid atmosphere of 5% CO_2_ at 37°C. The siRNAs used to target lncRNAs were synthesized by GenePharma Co., Ltd (Shanghai China). The siRNA sequences are listed in **Table S1**. Cells (2.0 × 10^5^ cells per well) were seeded in 6-well plates 24 h before transfection. The siRNAs were transfected using Lipofectamine 3000 (ThermoFisher Scientific, USA) according to the manufacturer’s protocol. A qPCR analysis was performed to determine the relative expression levels of the target lncRNAs. Expression levels of 18S rRNA were used as internal controls. The primer sequences used are listed in **Table S2**.

### Cell proliferation, migration, and invasion assays

Cell proliferation was assessed using the EdU staining kit (RiboBio, Guangzhou, China) according to the manufacturer’s protocol. Trans-well migration and invasion assays were performed as previously described with modification (27). Briefly, for the migration assay, cells were seeded in 24-well trans-well plates (Falcon, USA) with a density of 1 × 10^6^ cells per well in 100 µL of serum-free media. Meanwhile, for the invasion assay, 1 × 10^5^ cells were seeded in each well of the 24-well trans-well plates coated with Matrigel (Corning, USA). After 24 h of incubation, the cells in the upper chamber of the membranes were removed using cotton swabs, then cells in the lower chamber were stained with crystal violet (Amresco, USA) for 10 min and washed with PBS.

## Results

### Determining the lncRNA expression profile of LSCC patients by RNA-seq

**Figure 1:**
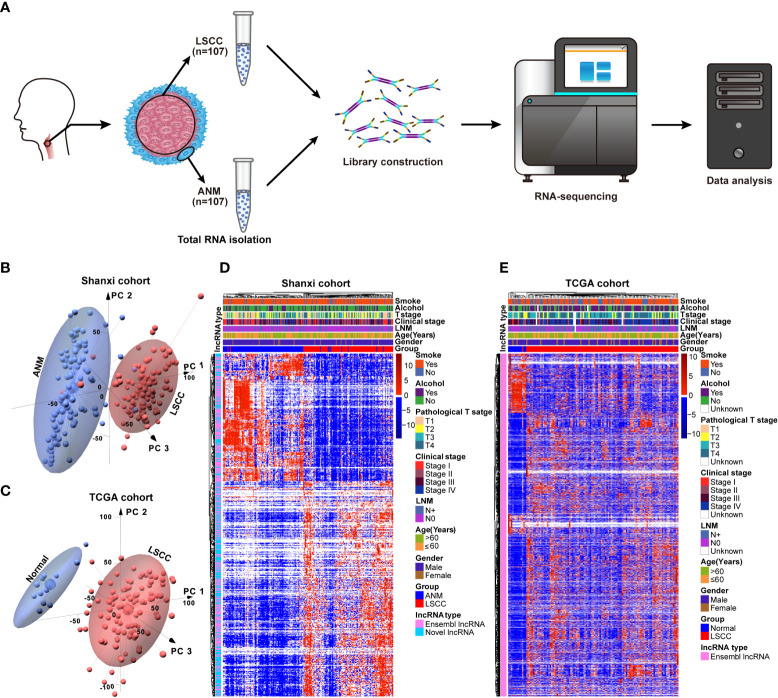
Construction of the lncRNAs expression profile in LSCC. **(A)** Schematic representation of the RNA-seq workflow using 107 LSCC and ANM tissues. **(B)** The PCA of lncRNAs between LSCC and ANM tissues in Shanxi cohort, red: LSCC tissue; blue: ANM tissue. **(C)** The PCA analysis of lncRNAs between LSCC and normal samples in the TCGA cohort, red: LSCC tissue; blue: normal tissue. **(D, E)** Heat map showing the correlation between lncRNAs expression levels and clinical features in the Shanxi **(D)** and TCGA **(E)** cohorts.

A total of 107 LSCC and paired ANM tissues were collected from 107 patients who were clinically diagnosed with LSCC to constitute the Shanxi cohort. The RNA samples from the Shanxi cohort were subjected to transcriptome sequencing (**Figure 1A**). Furthermore, the RNA-seq data of 111 LSCC patients were obtained from the TCGA database. The analysis workflow used in this study is shown in **Figure S1**. The PCA results showed that the lncRNA expression patterns in LSCC tissues are different from that of the paired ANM tissues (**Figure 1B**). This trend was further confirmed by the TCGA cohort (**Figure 1C**). Next, an unsupervised hierarchical clustering based on the lncRNA expression data and clinical features was performed. The results showed that numerous lncRNAs were differentially expressed between the LSCC and ANM/normal groups (**Figures 1D, E**).

### Dysregulated lncRNAs in LSCC are involved in tumorigenesis-related processes and pathways

**Figure 2:**
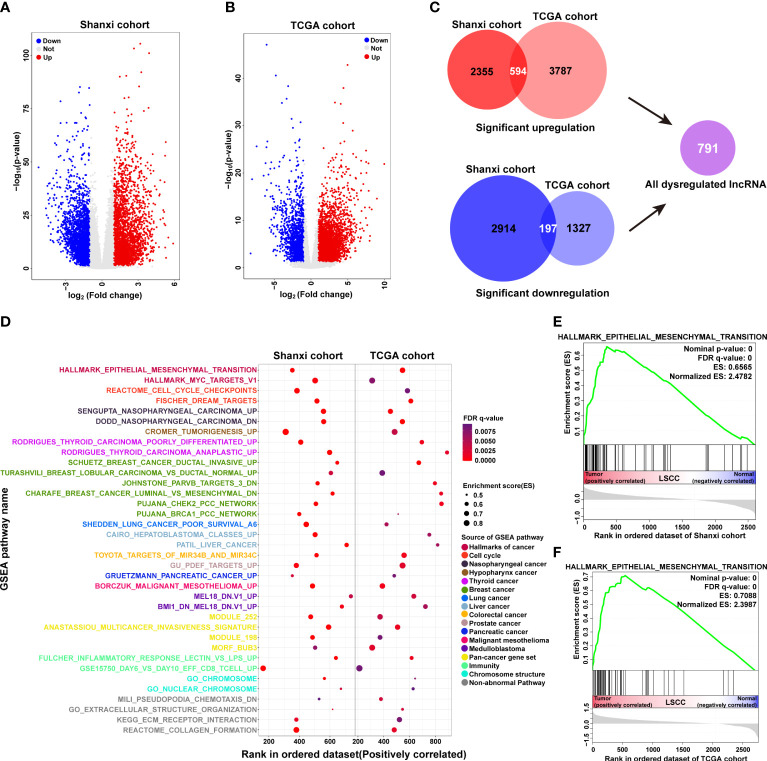
Functional annotation of significantly dysregulated lncRNAs in LSCC. **(A, B)** Volcano plots showing differentially expressed lncRNAs in the Shanxi **(A)** and TCGA **(B)** cohorts. **(C)** Analysis of the common differentially expressed lncRNAs in both Shanxi and TCGA cohorts, red: significantly upregulated lncRNAs; blue: significantly downregulated lncRNAs. **(D)** Functional annotation of the common differentially expressed lncRNAs in both Shanxi and TCGA cohorts by GSEA. **(E, F)** Enrichment plot of the EMT pathway in the Shanxi **(E)** and TCGA **(F)** cohorts.

Based on a threshold of p< 0.05 and |fold-change| ≥ 2, a total of 6,060 differentially expressed lncRNAs were identified in the Shanxi cohort. Out of these, 2,949 were upregulated and 3,111 were downregulated in LSCC tissues compared with ANM tissues (**Figure 2A** and **Table S3**). Meanwhile, in the TCGA cohort, 4,381 upregulated and 1,524 downregulated lncRNAs were identified in LSCC samples compared to normal samples (**Figure 2B** and **Table S4**). Using Venn analysis, we obtained 594 upregulated and 197 downregulated lncRNAs from both cohorts (**Figure 2C**), which were named as significantly dysregulated lncRNAs (SDLs). Next, we predicted the biological processes and pathways associated with the SDLs using GSEA. Results showed 36 mutual biological processes and pathways (FDR q-value ≤ 0.01) in both cohorts, including terms such as epithelial to mesenchymal transition (EMT), nasopharyngeal carcinoma, cell cycle checkpoints, tumorigenesis, thyroid carcinoma poorly differentiated, and malignant mesothelioma (**Figure 2D**). These suggested that the SDLs were involved in the regulation of multiple tumorigenesis-related processes and pathways.

EMT plays an important role in the development and metastasis of cancer (28, 29). We observed that the SDLs in both LSCC cohorts were significantly enriched in EMT (**Figures 2E, F**), suggesting that the dysregulated lncRNAs may affect LSCC invasion and metastasis by regulating the EMT process.

### Screening for lncRNAs associated with the OS of LSCC patients

**Figure 3:**
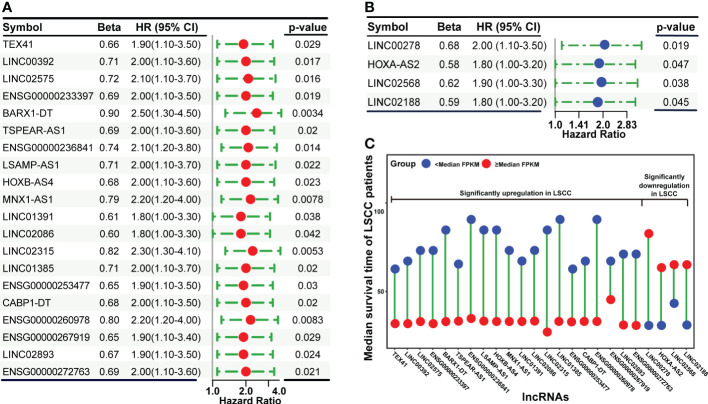
Cox proportional hazards regression analysis of significantly dysregulated lncRNAs using patients’ clinical information in the TCGA dataset. **(A)** Forest plot showing the upregulated lncRNAs that are significantly associated with OS. **(B)** Forest plot showing the downregulated lncRNAs that are significantly associated with OS. **(C)** The correlation between lncRNAs expression levels and median survival time of LSCC patients.

To screen for lncRNAs that are significantly associated with the OS of LSCC patients, all SDLs and prognosis-related clinical information of TCGA patients were subjected to Cox proportional hazards regression analysis. Using the criterion of p-value ≤ 0.05, 20 upregulated (TEX41, LINC00392, LINC02575, ENSG00000233397 (named as ENSG233397 thereafter), BARX1-DT, TSPEAR-AS1, ENSG00000236841, LSAMP-AS1, HOXB-AS4, MNX1-AS1, LINC01391, LINC02086, LINC02315, LINC01385, ENSG00000253477, CABP1-DT, ENSG00000260978, ENSG00000267919, LINC02893 (also known as AL513318.2), and ENSG00000272763) and 4 downregulated (LINC00278, HOXA-AS2, LINC02568, and LINC02188) lncRNAs were obtained from the univariate Cox analysis (**Figures 3A, B**). The association between the expression levels of these 24 lncRNAs and OS of LSCC patients was verified using K-M survival analysis (**Figures S2, S3**). Results showed that LSCC patients with high expression levels (≥ median FPKM) of each of the 20 upregulated lncRNAs had a shorter median OS time than that of the low expression group (< median FPKM). In contrast, LSCC patients with high expression levels of each of the 4 downregulated lncRNAs had a longer median OS time than that of the low expression group (**Figure 3C**).

### Construction and validation of a seven-lncRNAs panel for predicting the prognosis of LSCC patients

**Figure 4:**
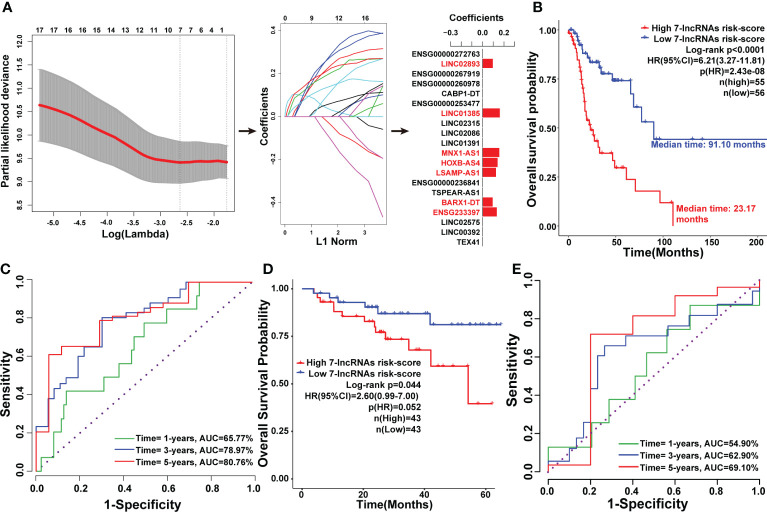
Construction of the seven-lncRNAs signature for predicting prognosis of LSCC patients. **(A)** Schematic representation of the LASSO algorithm workflow for the construction of the seven-lncRNAs signature. **(B)** K-M survival analysis showing the effect of the seven-lncRNAs signature risk score in the OS of LSCC patients in TCGA cohort. **(C)** The ROC curve for the seven-lncRNAs signature risk score in predicting the 1-, 3-, and 5-years OS of LSCC patients in TCGA cohort. **(D)** K-M survival analysis showing the efficiency of the seven-lncRNAs signature risk score in the OS of LSCC patients in 86 LSCC samples. **(E)** The ROC curve for the seven-lncRNAs signature risk score in predicting the 1-, 3-, and 5-years OS of LSCC patients in 86 LSCC samples.

To construct a model for predicting the prognosis of LSCC patients, LASSO regression analysis was used to narrow down the candidate lncRNAs based on the output of the Cox analysis. This led to the identification of seven lncRNAs including ENSG233397, BARX1-DT, LSAMP-AS1, HOXB-AS4, MNX1-AS1, LINC01385, and LINC02893 (**Figure 4A**). These seven lncRNAs were significantly upregulated in LSCC tissues compared to normal counterparts in both the Shanxi and TCGA cohorts (**Figures S4, S5**). Combining the relative expression of the lncRNAs and their corresponding coefficients, the risk score for the seven-lncRNAs of each patient was calculated as follows: risk score = 0.1331 × ${\text{e}}_{\mathrm{ENSG}233397}$ + 0.0940 × $e_{\mathrm{BARX}1-\mathrm{DT}}$ + 0.1254 × $e_{\mathrm{LSAMP}-\mathrm{AS}1}$ + 0.1400 × $e_{\mathrm{HOXB}-\mathrm{AS}4}$ + 0.1560 × $e_{\mathrm{MNX}1-\mathrm{AS}1}$ + 0.1611 × $e_{\mathrm{LINC}01385}$ + 0.0959 × $e_{\mathrm{LINC}02893}$ . The risk scores of the seven-lncRNAs panel were significantly associated with poor OS (HR:6.21 [3.27-11.81], p<0.0001), DSS (HR:4.34 [1.83-10.26], p=0.0008), and PFI (HR:3.78 [1.92-7.43], p=0.0001) in TCGA cohort. Based on the median value of the risk scores, all patients in the TCGA cohort were divided into high and low risk groups. K-M survival analysis revealed that LSCC patients in the high risk group had shorter median OS (23.17 months), DSS (48.87 months), and PFI (19.23 months) than those in the low risk group (OS: 91.10 months, DSS: 213.90 months, and PFI: 213.90 months; **Figures 4B**, **S6A, C**).

Time-dependent ROC curves were drawn to assess the prognostic ability of the seven-lncRNAs risk scores. In the time frame of 1-, 3-, and 5-years, the AUC index of the seven-lncRNAs risk score for OS were 65.77%, 78.97%, and 80.76%; for DSS were 58.47%, 75.99%, and 76.55%; and for PFI were 63.20%, 73.41%, and 74.70%, respectively (**Figures 4C**, **S6B, D**). To validate the seven-lncRNAs prognostic model, the expression levels of these lncRNAs in 86 LSCC samples were determined by qPCR, then the association between seven-lncRNAs risk score and overall survival of the 86 LSCC patients was evaluated by Kaplan-Meier analysis. As expected, results further confirmed that the seven-lncRNAs model was able to predict prognosis of LSCC patients (**Figure 4D**). Furthermore, time-dependent ROC curves analysis showed that the AUC index of the seven-lncRNAs risk score of the 86 LSCC patients were 54.90%, 62.90%, and 69.10% in the time frame of 1-, 3-, and 5-years for OS (**Figure 4E**).

**Figure 5:**
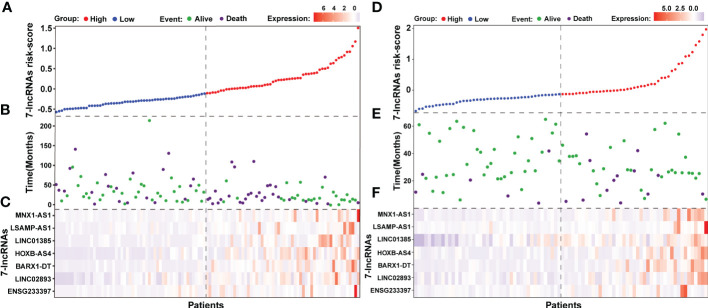
The outcome of patients and lncRNAs expression distribution between the seven-lncRNAs high and low risk groups. **(A)** The seven-lncRNAs signature risk scores ranked from low to high in TCGA cohort. **(B)** Survival status and duration of patients in TCGA cohort. **(C)** Heatmap showing the lncRNAs expression between the high and low risk groups in TCGA cohort. **(D)** The seven-lncRNAs signature risk scores ranked from low to high in 86 LSCC samples. **(E)** Survival status and duration of patients in 86 LSCC samples. **(F)** Heatmap showing the lncRNAs expression between the high and low risk groups in 86 LSCC samples.

We also noted that LSCC patients in the low risk group had a lower mortality rate (20/55 patients, 36.36%) than those in the high risk group (30/56 patients, 53.57%; **Figures 5A, B**). Furthermore, the identified seven lncRNAs had higher expression levels in patients of the high risk group than those in the low risk group (**Figure 5C**). Further analysis of the seven-lncRNAs data of the 86 LSCC patients showed that patients in high risk group had a higher mortality rate (13/43 patients, 30.23%) than those in the low risk group (6/43 patients, 13.95%), and patients in high risk group had higher expression levels of seven lncRNAs (**Figures 5D–F**).

**Figure 6:**
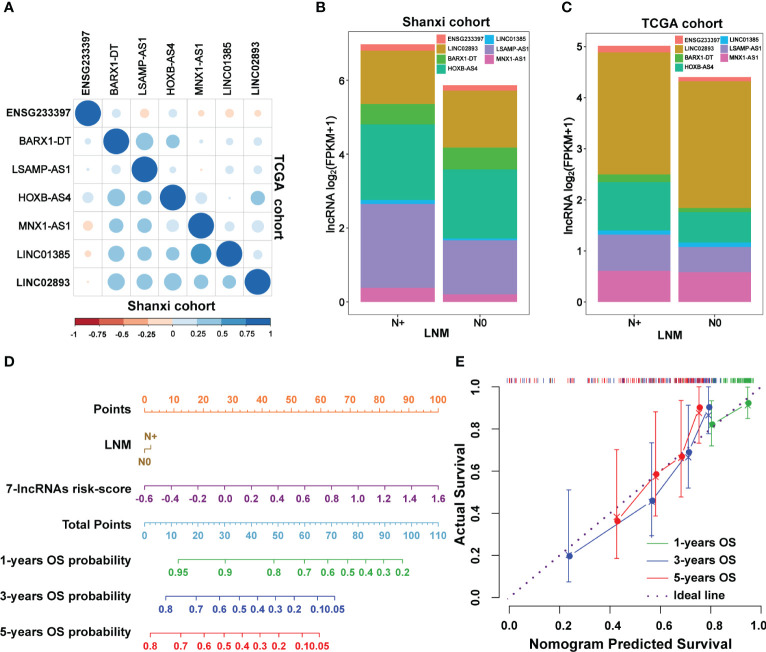
The correlation between lymph node metastasis (LNM) and the seven-lncRNAs signature. **(A)** The Pearson’s correlation analysis showing the relationship of the seven lncRNAs with each other in the Shanxi and TCGA cohorts. **(B, C)** Association between the expression levels of the seven lncRNAs and lymph node metastasis in the Shanxi **(B)** and TCGA **(C)** cohorts. **(D)** The constructed prognostic nomogram which consists of the seven-lncRNAs signature risk score and lymph node metastasis information for predicting the 1-, 3-, and 5-years OS of LSCC patients. **(E)** Calibration curves of the prognostic nomogram estimating the probability of OS in 1-, 3-, and 5-years. LNM, lymph node metastasis; N0, LSCC without lymph node metastasis; N+, LSCC with lymph node metastasis.

To analyze the interconnections among seven lncRNAs, a total of 18 miRNAs was predicted based on ceRNA hypothesis (**Figure S7A**). LINC01385 and BARX1-DT had a common targeted miRNA named as miR-4739 (**Figure S7B**). ceRNA network analysis revealed that direct or indirect regulatory relationships were existed among these seven lncRNAs (**Figure S7C**). The Pearson’s correlation analysis of the seven lncRNAs versus each other in the Shanxi and TCGA cohorts is seen in **Figure 6A**. LNM is a predominant independent factor associated with the mortality rate in LSCC (30). We found that the seven lncRNAs had relatively higher expression levels in patients with LNM than those of non-metastatic patients in both cohorts (**Figures 6B, C**, **S8**). To further improve the predictive accuracy of our model, a nomogram for OS prediction was constructed by integrating the risk score and clinical features of LNM. As shown in **Figure 6D**, the upward vertical line can be respectively drawn from the line of the risk score and LNM to the line where their respective points can be calculated. Based on the sum of points of the seven-lncRNAs risk score and LNM, a downward vertical line can be drawn from the line of the total points to the line of the 1-, 3-, or 5-year OS probability to respectively calculate the OS probability. Additionally, the calibration curves for the prognostic nomogram estimating the probability of 1-, 3-, and 5-years OS performed well with the ideal model (**Figure 6E**). Decision curve analysis of the prognostic nomogram for 1-, 3-, and 5-years OS risk showed that the model has a net benefit of risk stratification in the gradient prediction time (**Figure S9**). Collectively, these findings demonstrated that a seven-lncRNAs panel for predicting the prognosis of LSCC patients was constructed using an integrated analysis of our large-scale RNA-seq data and the TCGA data.

### Knockdown of each of the seven lncRNAs inhibits the proliferation, migration, and invasion of LSCC cells

**Figure 7:**
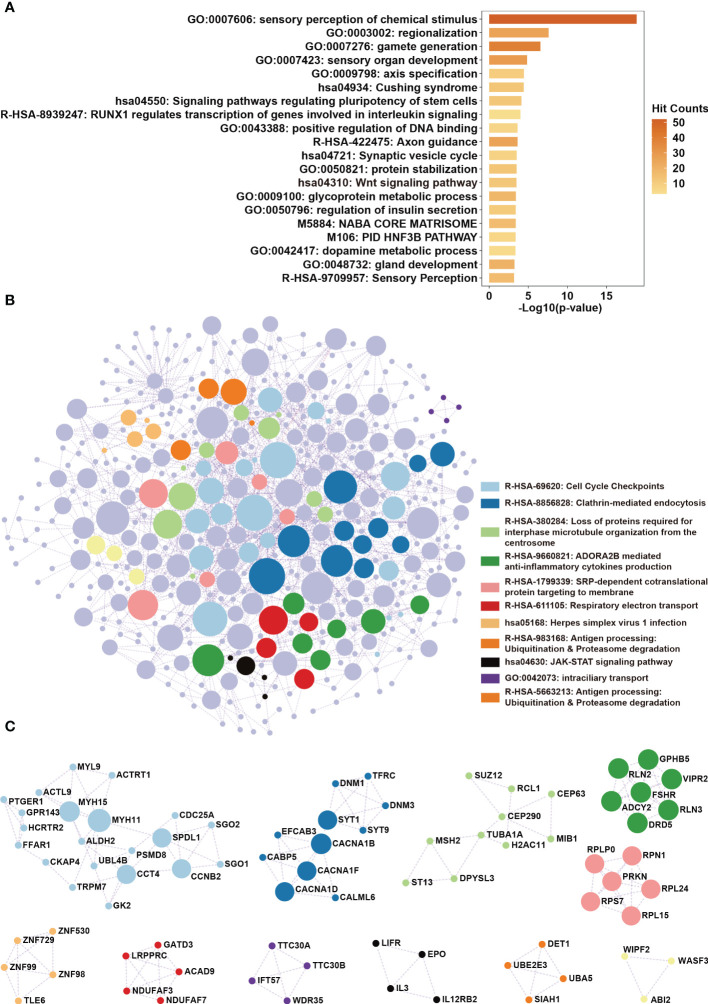
Functional annotation of the lncRNAs in the seven-lncRNAs panel. **(A)** Protein-coding genes whose expression levels were correlated with lncRNAs ENSG233397, BARX1-DT, LSAMP-AS1, HOXB-AS4, MNX1-AS1, LINC01385, and LINC02893 were obtained by correlation analysis. KEGG pathway and biological process enrichment analysis were performed using Metascape. **(B)** Protein-protein interaction enrichment analysis involving the protein products of the identified genes. **(C)** Protein complexes identified in protein-protein interaction enrichment analysis were highlighted.

We sought to determine whether the lncRNAs are involved in regulating LSCC progression. We used the protein-coding genes whose expression levels significantly correlated with the seven lncRNAs for functional annotation through Metascape (**Table S5**). Results showed that the seven lncRNAs were mainly involved in the Wnt signaling pathway and pathways regulating the pluripotency of stem cells (**Figure 7A**). Furthermore, protein-protein interaction enrichment analysis revealed that the seven lncRNAs were also involved in cell cycle checkpoints and the JAK-STAT signaling pathway (**Figures 7B, C**). Since these pathways are known to contribute to cellular growth and differentiation, we hypothesized that these lncRNAs may regulate the progression of LSCC.

**Figure 8:**
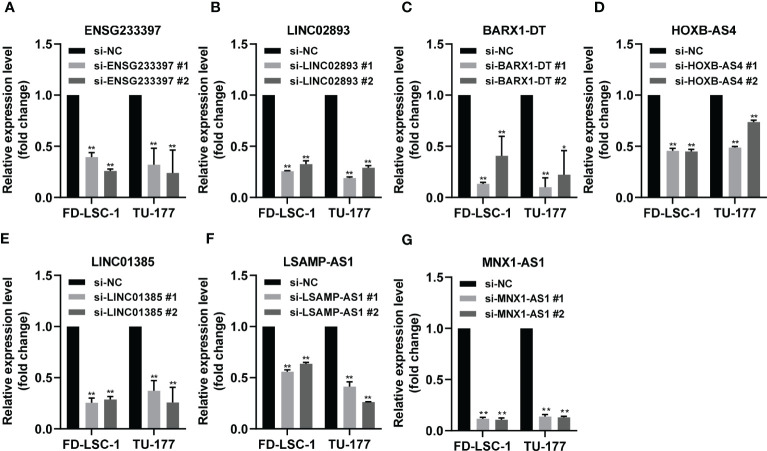
Validation of the knockdown efficiency by qPCR analysis. **(A–G)** FD-LSC-1 and TU-177 cells were transfected with siRNAs targeting each of the lncRNAs ENSG233397 **(A)**, LINC02893 **(B)**, BARX1-DT **(C)**, HOXB-AS4 **(D)**, LINC01385 **(E)**, LSAMP-AS1 **(F)**, and MNX1-AS1 **(G)**, respectively. After 48 h of transfection, the expression levels of each of the lncRNAs were determined by qPCR analysis. All experiments were repeated three times, data are shown as mean ± SD. ** p < 0.01.

**Figure 9:**
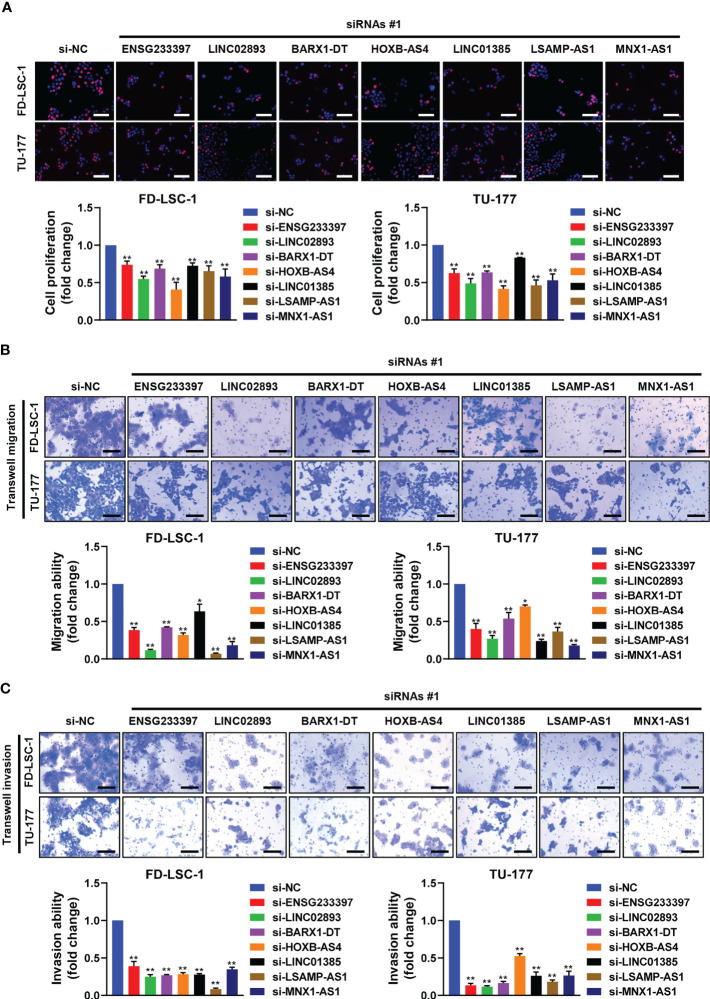
Effects of the seven lncRNAs on the cell proliferation, migration, and invasion of LSCC cells. FD-LSC-1 and TU-177 cells were transfected with siRNAs targeting the lncRNAs ENSG233397, BARX1-DT, LSAMP-AS1, HOXB-AS4, MNX1-AS1, LINC01385, and LINC02893, respectively. **(A)** Cell proliferation was assessed by EdU staining. Scale bar, 100 μm. **(B)** Cell migration ability was assessed by trans-well migration assay. Scale bar, 200 μm. **(C)** Cell invasion ability was assessed by trans-well invasion assay. Scale bar, 200 μm. All experiments were repeated three times, data are shown as mean ± SD. * *p* < 0.05, ** *p* < 0.01.

To investigate the functional roles of each of the seven lncRNAs in our model, we performed siRNA-mediated knockdown targeting the identified lncRNAs in LSCC cell models. Our qPCR analysis confirmed that the expression levels of the seven lncRNAs decreased significantly in the LSCC cell lines FD-LSC-1 and TU-177 after siRNAs transfection (**Figure 8**). The EdU staining results showed that knocking down ENSG233397, BARX1-DT, LSAMP-AS1, HOXB-AS4, MNX1-AS1, LINC01385, and LINC02893, separately, inhibits the proliferation of LSCC cells to different degrees (**Figures 9A**, **S10A**). In addition, we found that knockdown of each of the seven lncRNAs resulted in significantly decreased migration and invasion abilities of LSCC cells (**Figures 9B, C**; **S10B, C**). Collectively, our findings suggest that the lncRNAs used in our model promote the proliferation, migration, and invasion abilities of LSCC cells *in vitro*.

## Discussion

Although increasing clinical studies and advancements in treatment have been done in recent years, satisfactory therapeutic strategies and accurate prognosis predictors for LSCC have not yet been developed (31). To date, conventional cytopathology and histopathology analysis are still the sole methods for the clinical diagnosis of LSCC. Previous studies have identified several potential pathologic factors contributing to LSCC, including smoking, alcohol drinking, air pollution, HPV infection, and sex hormone receptor levels (32, 33). However, the detailed mechanism underlying tumorigenesis and progression of LSCC remains unclear. In addition, no molecular biomarkers are currently used for the diagnosis and assessment of progression as well as prognosis in LSCC (34). Therefore, the identification of molecular biomarkers to diagnose LSCC at an early stage and predict the disease progression and prognosis is urgent for clinicians and researchers.

LncRNAs have shown obvious advantages as biomarkers of human disease, particularly for the diagnosis and prognosis of cancers (7, 35): (1) The large number of lncRNAs provides a wide range of options for finding new biomarkers; (2) lncRNA acts in various forms, which reflects the complexity of gene regulatory network in cancer; (3) lncRNA exists in various materials, such as tissue, peripheral blood, exosome, thereby providing a variety of detection and sampling methods for diagnosis; (4) lncRNA expression is tissue and spatio-temporal specific, which is of great value in diagnosis, pharmacodynamic evaluation, and prognosis evaluation. Hence, the identification of lncRNA biomarkers is of great significance for the diagnosis and assessment of the prognosis of LSCC.

Recently, several lncRNAs have been identified as potential biomarkers for the diagnosis and prognosis of LSCC. Liang et al. reported that the high expression of the lncRNA snaR was associated with the progression and poor prognosis of LSCC (36). In addition, the high expression of LINC01194 has been shown to be negatively correlated with the clinical outcome of LSCC patients (37). Moreover, the low expression of lncRNA SBF2-AS1 was correlated to lymph node metastasis, advanced clinical stage, as well as poor prognosis of LSCC (38). Despite of these findings, no lncRNA biomarker was used to predict the prognosis of LSCC clinically. Hence, identifying more lncRNA biomarkers with high specificity for evaluating LSCC progression and prognosis is needed. In the identification of biomarkers, large sample sizes are used to overcome substantial inter-study heterogeneity and enhance reliability (39, 40). In the current study, RNA-seq data of 107 LSCC and paired ANM tissues were integrated with the TCGA-published LSCC RNA-seq data to identify differentially expressed lncRNAs between LSCC and normal tissues. To the best of our knowledge, the sample size used in this study is much larger than that of previous studies, which makes our results more robust and reliable.

Pathway analysis revealed that dysregulated lncRNAs in LSCC were involved in cancer-related biological processes and pathways, such as epithelial to mesenchymal transition, nasopharyngeal carcinoma, cell cycle checkpoints, tumorigenesis, thyroid carcinoma poorly differentiated, and malignant mesothelioma. These findings indicate that the dysregulation of lncRNAs is crucial in regulating LSCC phenotypes. Moreover, these lncRNAs may regulate LSCC progression through the above-mentioned pathways.

Recent studies reported that multi-genes signature has a more precise predictive performance than using a single biomarker (36). We constructed a seven-lncRNAs panel (ENSG233397, BARX1-DT, LSAMP-AS1, HOXB-AS4, MNX1-AS1, LINC01385, and LINC02893) that has high specificity and sensitivity for predicting prognosis of LSCC. MNX1-AS1 is a well-known oncogenic lncRNA in multiple types of cancers such as gastric cancer (41), colorectal cancer (42), and hepatocellular carcinoma (43). Liu et al. reported that high expression of LSAMP-AS1 was associated with the poor prognosis of LSCC (44). High expression of LINC01385 was correlated with advanced clinical features and poor prognosis of nasopharyngeal carcinoma (NPC), and knockdown of LINC01385 inhibited the proliferation and invasion abilities of NPC cells (45). Moreover, upregulation of LINC02893 in non-small cell lung cancer is associated with poor prognosis (46). Based on the bioinformatic analysis results, we speculated that there are potential mutual regulatory relationships among these seven lncRNAs. On the one hand, a lot of mRNAs was regulated by shared lncRNAs, these mRNAs may affect expression levels of these lncRNAs in turn. On the other hand, some of the seven lncRNAs have common target miRNA, for example, both LINC01385 and BARX1-DT target miR-4739.

In this study, we further explored the functional roles of the seven lncRNAs used in our model. Our data revealed that the knockdown of each of the seven lncRNAs suppressed the cell proliferation, migration, and invasion abilities of LSCC cells *in vitro*. The downregulation of the seven lncRNAs contributes to the suppression of proliferation, migration, and invasion of LSCC cells, suggesting that these lncRNAs act as oncogenes in LSCC. Therefore, the seven lncRNAs may be potential targets for LSCC therapy. Further studies to uncover the molecular mechanisms underlying the activities of these lncRNAs in regulating the development of LSCC are required in the future.

In conclusion, we developed a seven-lncRNAs signature for predicting the prognosis of LSCC patients based on RNA-Seq data of more than two hundred LSCC patients from two cohorts. Risk scores and prognostic nomogram generated using the seven-lncRNAs signature performed well in evaluating the survival probability of LSCC patients. Furthermore, the lncRNAs used in our model promote proliferation, migration, and invasion of LSCC cells *in vitro*. Our findings provide a new potential multi-lncRNAs signature for predicting the prognosis of LSCC patients. More importantly, the RNA-seq dataset obtained in this study could be used as a resource of future basic and clinical cancer research.

## Data availability statement

The datasets presented in this study can be found in online repositories. The names of the repository/repositories and accession number(s) can be found below: Gene Expression Omnibus, accession numbers GSE127165 and GSE130605.

## Ethics statement

The studies involving human participants were reviewed and approved by the medical ethics committee of First Hospital of Shanxi Medical University. The patients/participants provided their written informed consent to participate in this study.

## Author contributions

Conceptualization: YW, CA, WG. Data curation: KW, ZG, WG, YW. Formal analysis: XWZ, WG, ZZ, XX, MM, QG, DW. Funding acquisition: YW, CA, XHZ, ZZ, WG. Investigation: XWZ, WG, ZZ, XX, MM, QG, DW. Methodology: KW, ZG, WG, LX, YW. Project administration: YW, CA, XHZ, WG. Resources: YW, ZG, WG. Supervision: YW, CA, XHZ. Validation: XWZ, WG, XX, MM, QG, DW. Visualization: XWZ, WG, XX, YW. Writing - review & editing: XWZ, WG, YW, CA, XHZ. All authors contributed to the article and approved the submitted version.
