# Supplementary material for: Identification of a seven-lncRNAs panel that serves as a prognosis predictor and contributes to the malignant progression of laryngeal squamous cell carcinoma
Source: Front Oncol. 2023 May 2;13:1106249. doi: 10.3389/fonc.2023.1106249 (PMC10188209; doi:10.3389/fonc.2023.1106249)
Supplement: Supplementary file 1 [file Table_1.doc]

| **siRNAs name** | **Sense (5'-3')** | **Antisense (5'-3')** |
| --- | --- | --- |
| si-ENSG233397 #1 | GCACAUAUGGAUAGAACAATT | UUGUUCUAUCCAUAUGUGCTT |
| si-ENSG233397 #2 | GCAAAUUAUGGCAUCCCUUTT | AAGGGAUGCCAUAAUUUGCTT |
| si-BARX1-DT#1 | GGAUUCGAGGAGAAGGGAATT | UUCCCUUCUCCUCGAAUCCTT |
| si-BARX1-DT#2 | GGCAGUACCUUCUCUAGAUTT | AUCUAGAGAAGGUACUGCCTT |
| si-MNX1-AS1#1 | GGUCGAACCUUAUCUGCUATT | UAGCAGAUAAGGUUCGACCTT |
| si-MNX1-AS1#2 | GCUACGUGAGUCUUGCAAATT | UUUGCAAGACUCACGUAGCTT |
| si-LINC01385#1 | CCAUGAACGUGAACAUGAACGUGUU | AACACGUUCAUGUUCACGUUCAUGG |
| si-LINC01385#2 | GGUGAAGUGGGUAUCUCCAUGUGAU | AUCACAUGGAGAUACCCACUUCACC |
| si-LSAMP-AS1#1 | GAGCAAACUAAUACAUUAAACAGAA | UUCUGUUUAAUGUAUUAGUUUGCUCAG |
| si-LSAMP-AS1#2 | GCAUUUCCAUCUGAGGUGGAAUGAC | GUCAUUCCACCUCAGAUGGAAAUGCAG |
| si-LINC02893#1 | CCUUCUCACUUUCUGCCUUTT | AAGGCAGAAAGUGAGAAGGTT |
| si-LINC02893#2 | CCGAGCCUGUUCAGUCUUUTT | AAAGACUGAACAGGCUCGGTT |
| si-HOXB-AS4#1 | UCAGUUUAGGGAAGAAGUUTT | AACUUCUUCCCUAAACUGATT |
| si-HOXB-AS4#2 | UCCACCACCGUCUCCUUAUTT | AUAAGGAGACGGUGGUGGATT |
| si-NC | UUCUCCGAACGUGUCACGUTT | ACGUGACACGUUCGGAGAATT |

**Table S1. Sequence of siRNAs targeting lncRNAs.**
