# Supplementary material for: Identification of a seven-lncRNAs panel that serves as a prognosis predictor and contributes to the malignant progression of laryngeal squamous cell carcinoma
Source: Front Oncol. 2023 May 2;13:1106249. doi: 10.3389/fonc.2023.1106249 (PMC10188209; doi:10.3389/fonc.2023.1106249)
Supplement: Supplementary file 2 [file Table_2.doc]

| **Primer name** | **Sense (5'-3')** | **Antisense (5'-3')** |
| --- | --- | --- |
| ENSG233397 | CCTCACAGAGAAGAAGGTTGTG | GGGTCATATGGAACCAAGGATAC |
| BARX1-DT | CTACGCTGAACTCCTCTCTTTG | ACTGAGTTCTCACAGTCTCTCT |
| MNX1-AS1 | CTCTGCAGGTCGAACCTTATC | AGTGTCTATCTGGAGGGTAGTT |
| LINC01385 | GGAACCTAGGCTATTCCTTGTG | CACTATCGCAATACAGCCTTCTA |
| LSAMP-AS1 | CAAGAGAGGCCTCAGAAGAATC | TAGTTTGCTAGGGTTGCCATAA |
| LINC02893 | CCTGTTGATAGTGAGGTACCAAG | CGAGACTCTTCGGAGGTTAATG |
| HOXB-AS4 | GCAACAAGAGAGGAGTCAAGAG | CAGCAAGTACCCGGCAATAA |
| 18S rRNA | CCTGGATACCGCAGCTAGGA | GCGGCGCAATACGAATGCCCC |

**Table S2. Primer sequence for qPCR analysis.**
