# Supplementary figures and images for: Identification of a seven-lncRNAs panel that serves as a prognosis predictor and contributes to the malignant progression of laryngeal squamous cell carcinoma
Source: Front Oncol. 2023 May 2;13:1106249. doi: 10.3389/fonc.2023.1106249 (PMC10188209; doi:10.3389/fonc.2023.1106249)

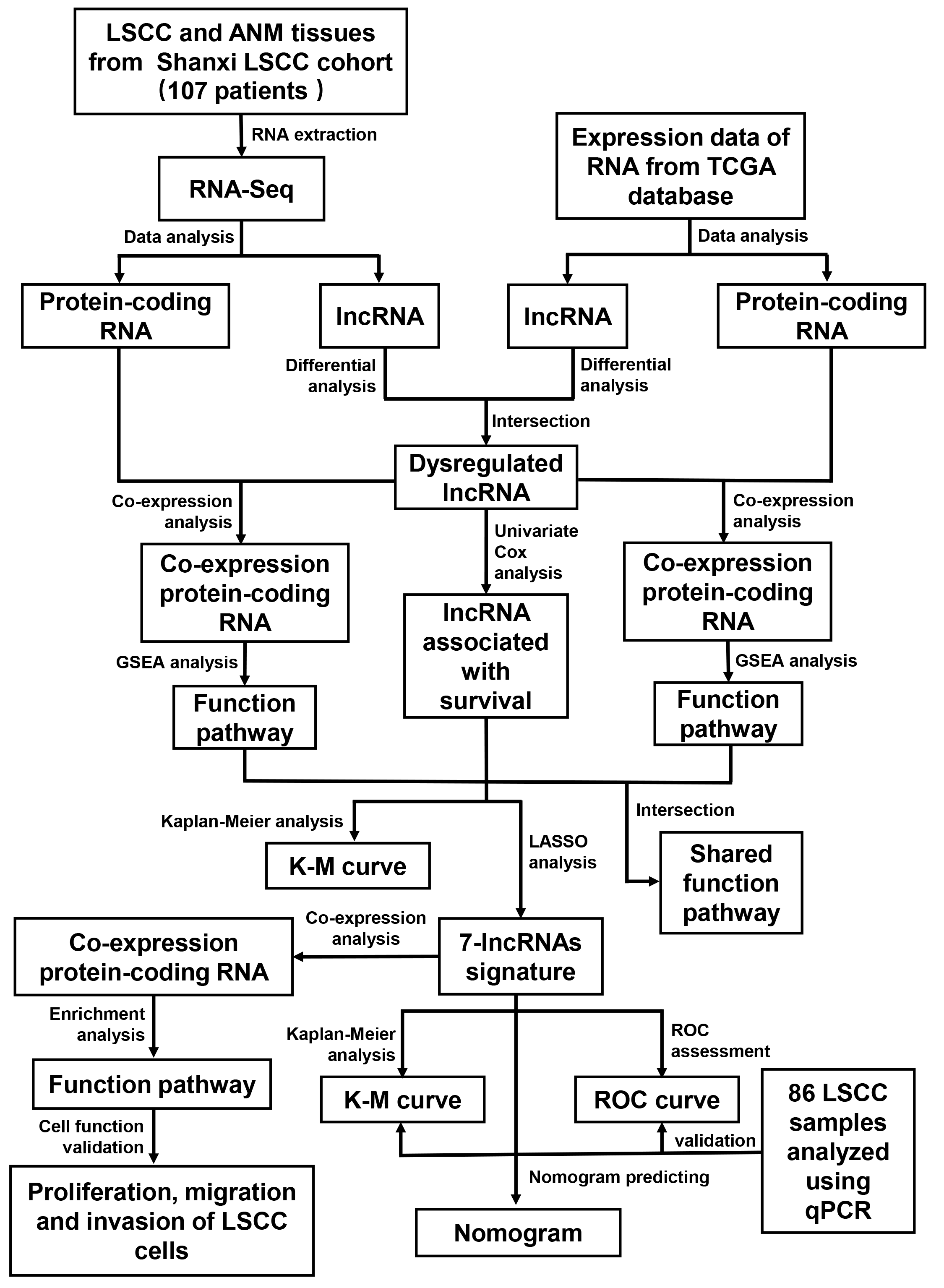

Supplement: Supplementary file 6 [file Image_1.tif]

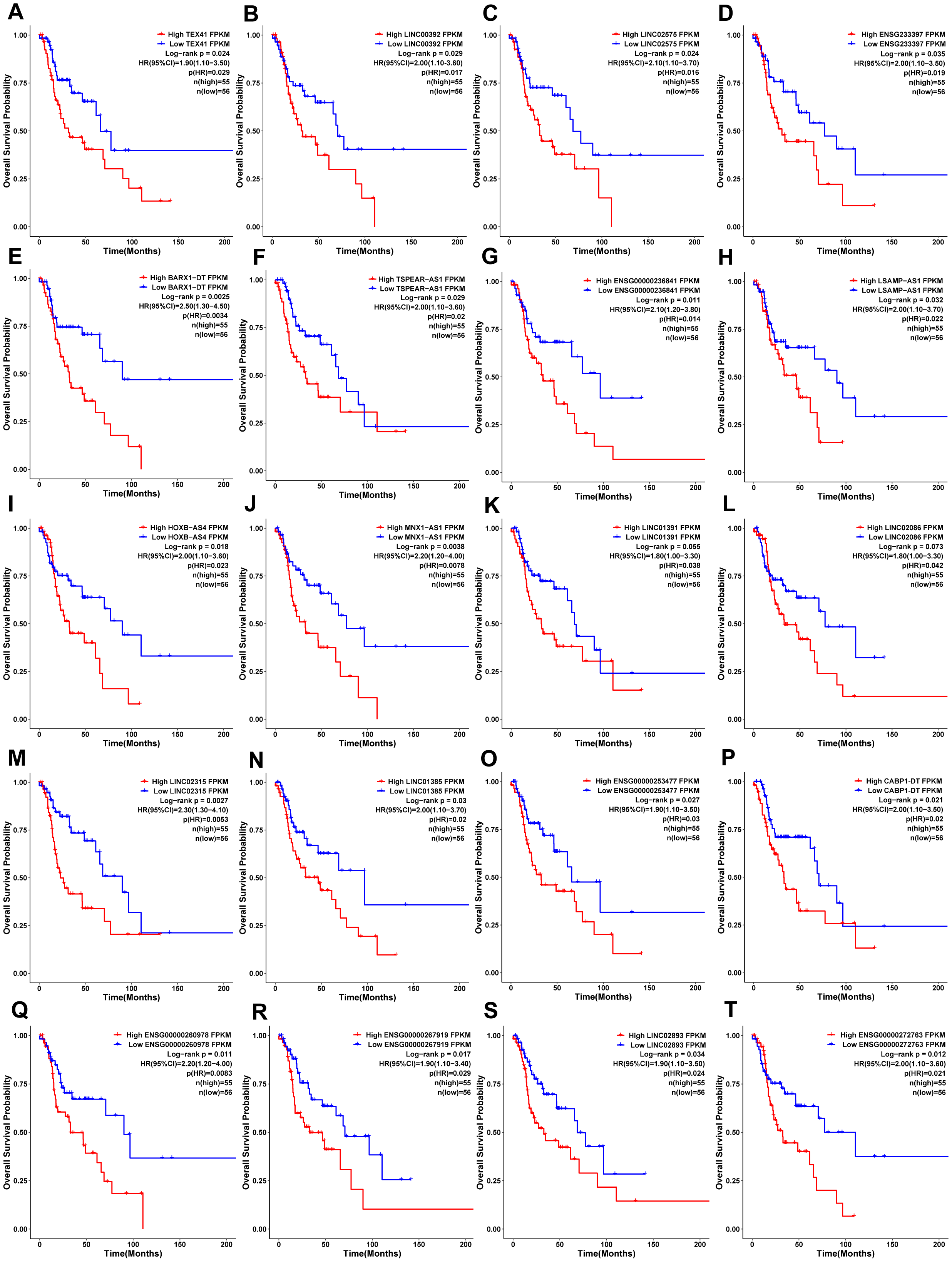

Supplement: Supplementary file 7 [file Image_2.tif]

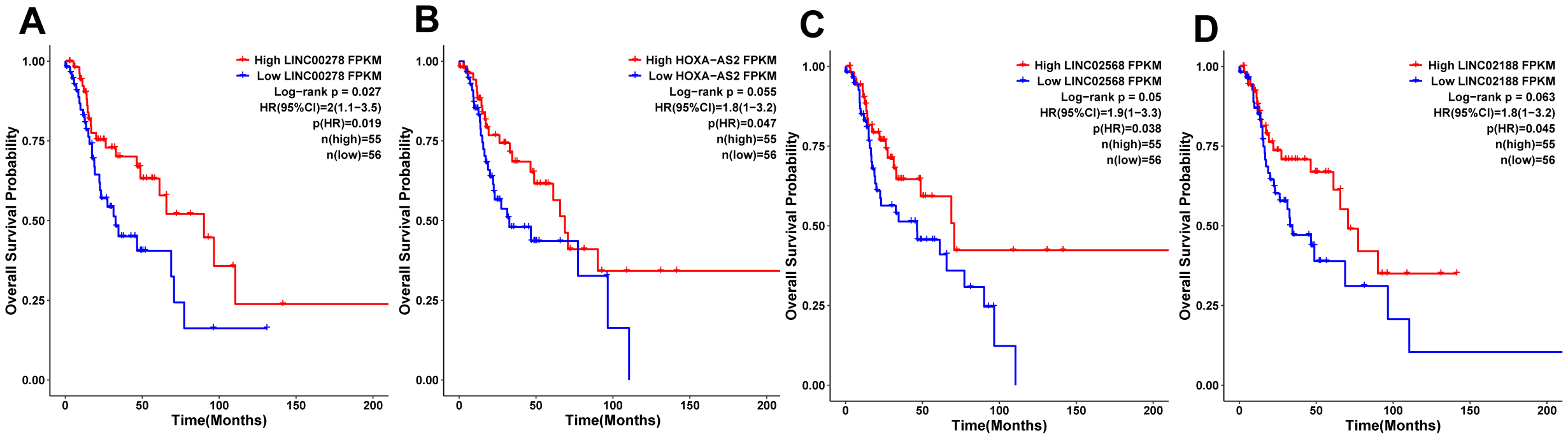

Supplement: Supplementary file 8 [file Image_3.tif]

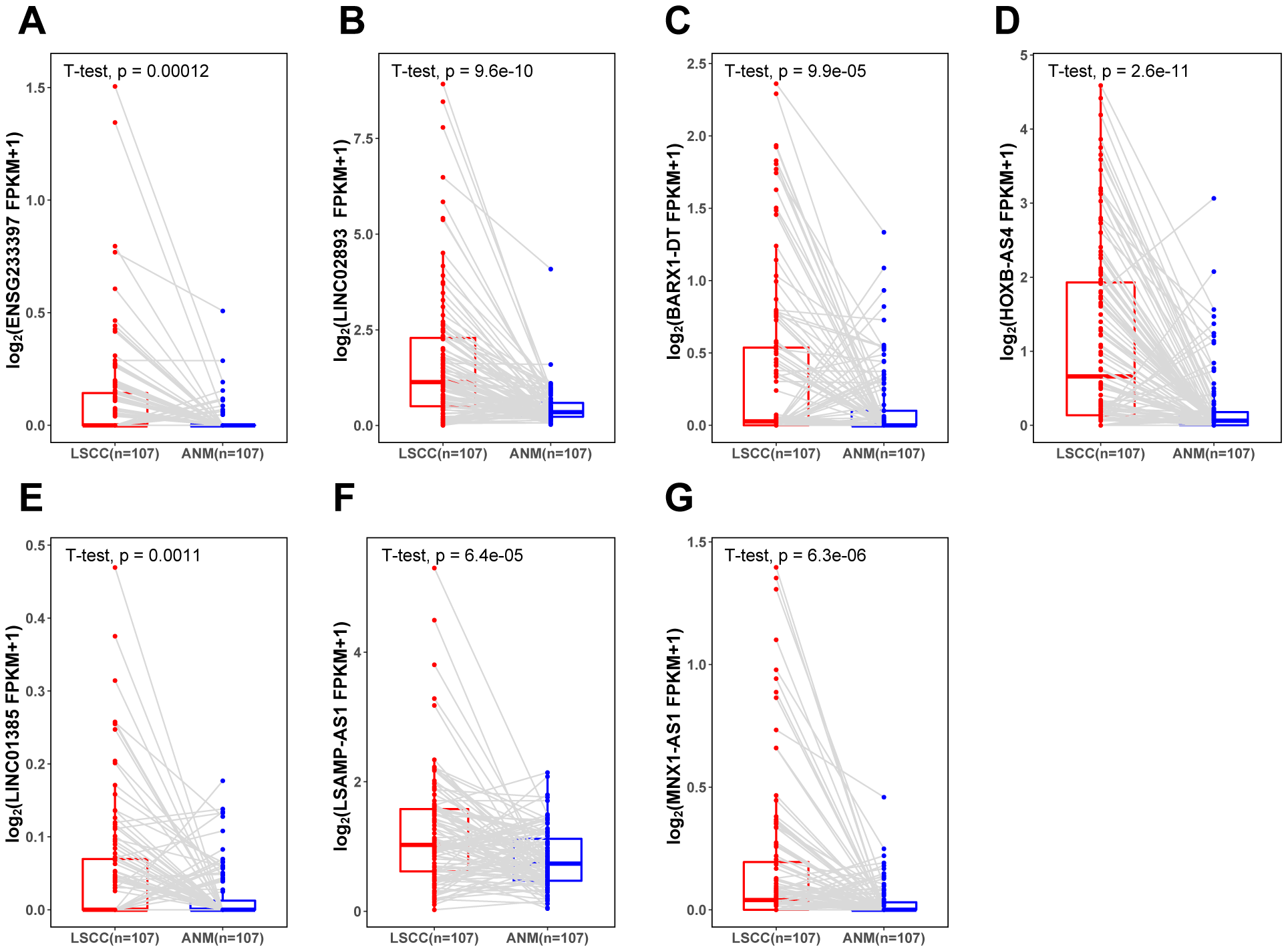

Supplement: Supplementary file 9 [file Image_4.tif]

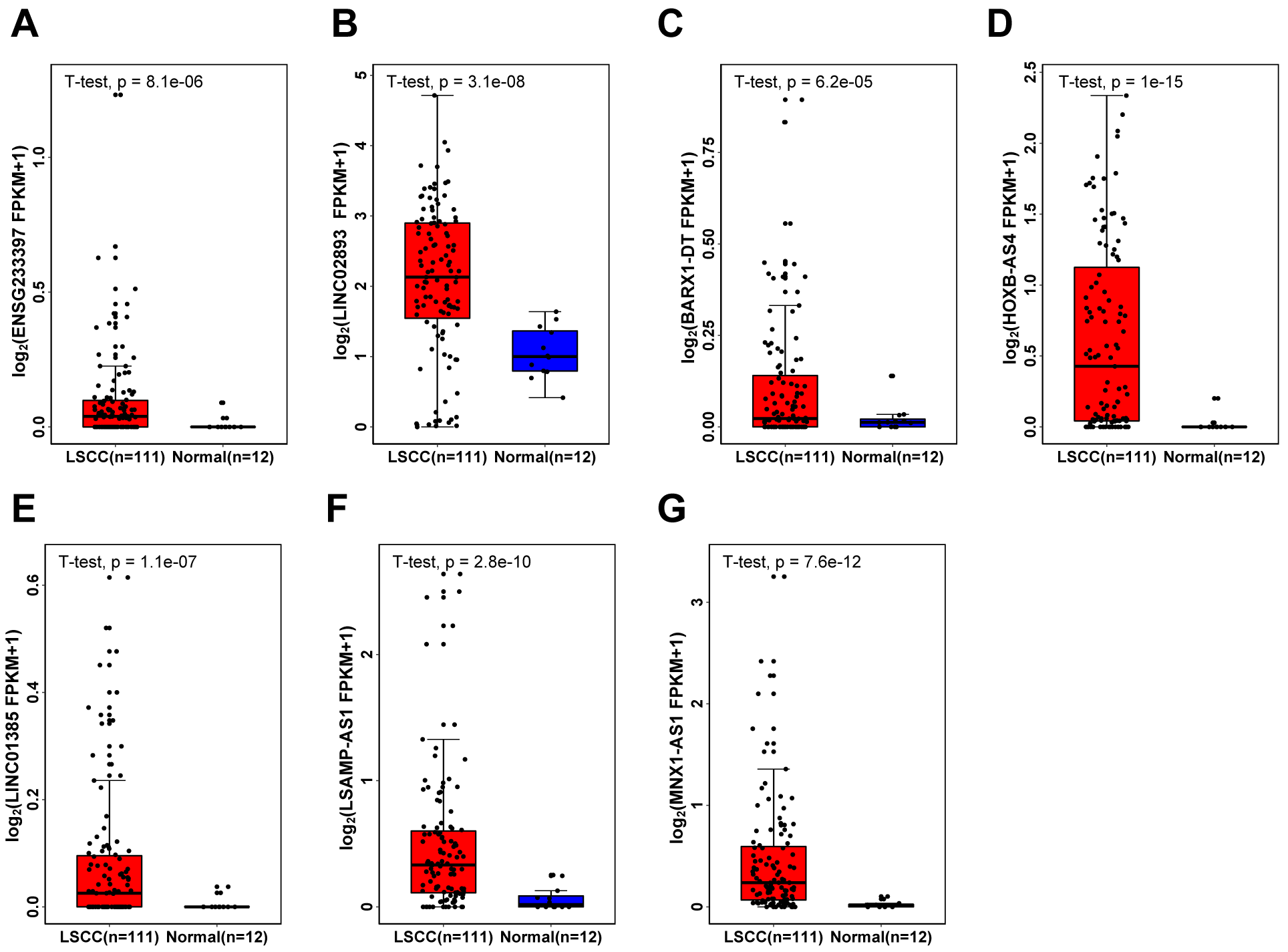

Supplement: Supplementary file 10 [file Image_5.tif]

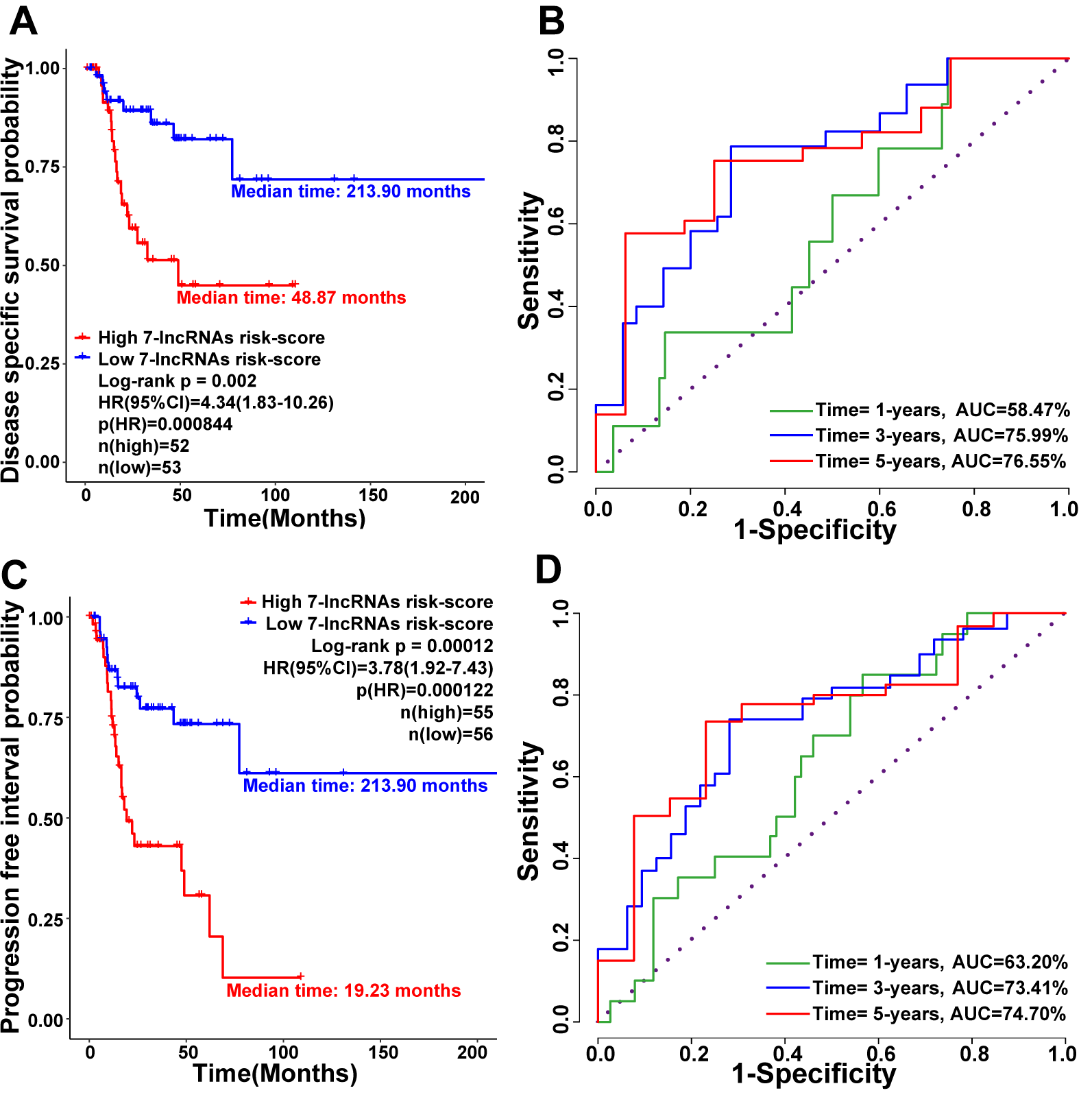

Supplement: Supplementary file 11 [file Image_6.tif]

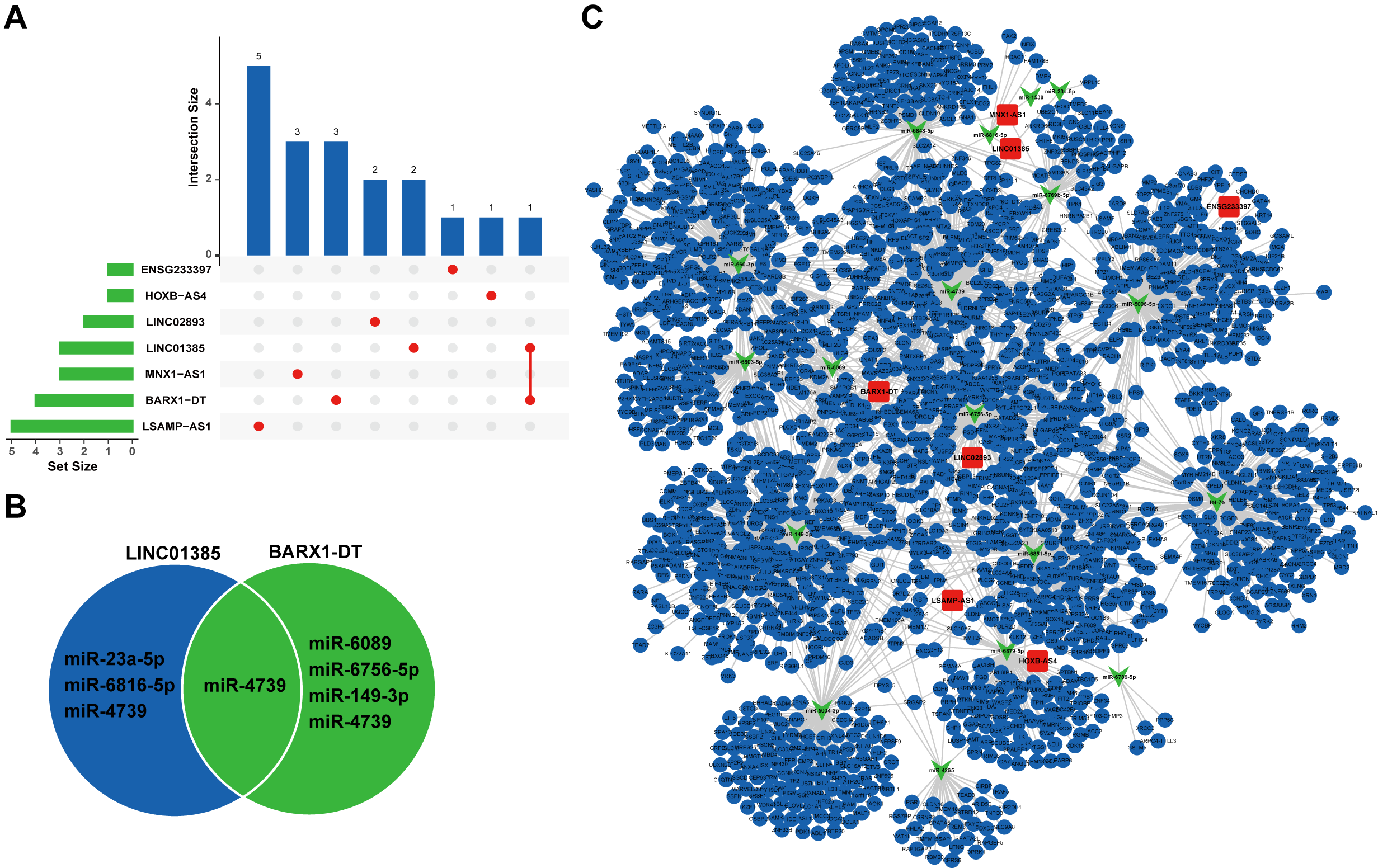

Supplement: Supplementary file 12 [file Image_7.tif]

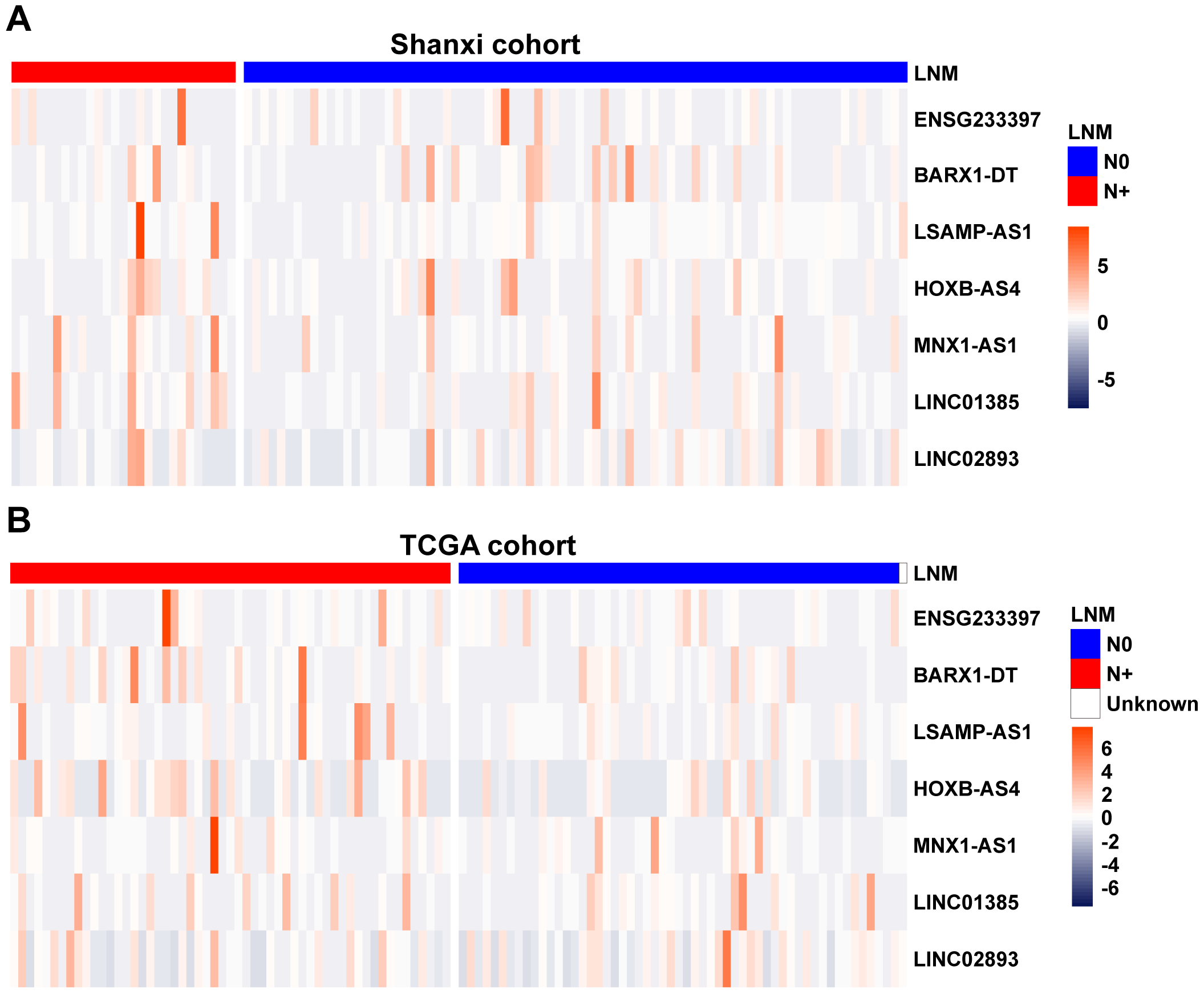

Supplement: Supplementary file 13 [file Image_8.tif]

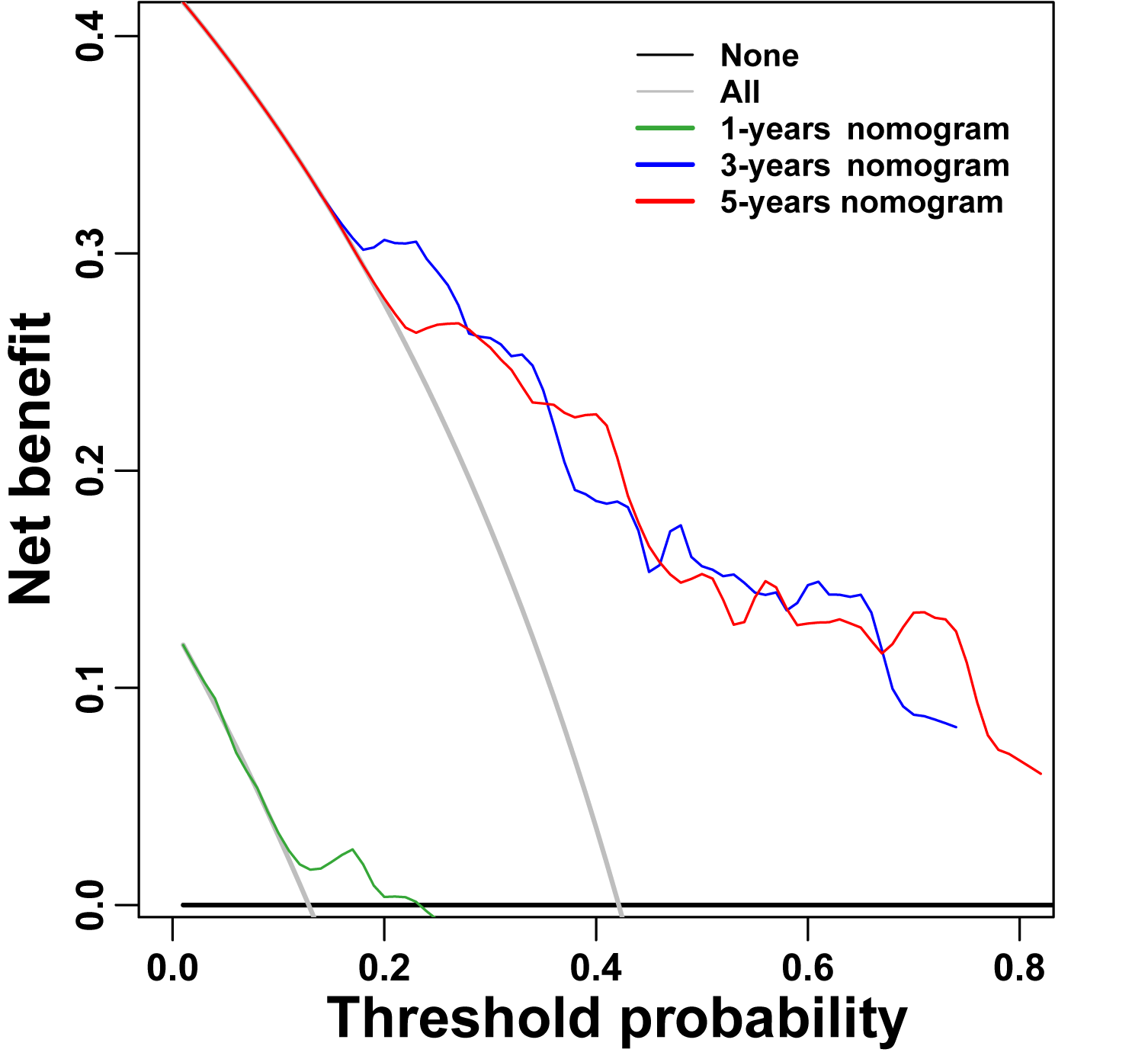

Supplement: Supplementary file 14 [file Image_9.tif]

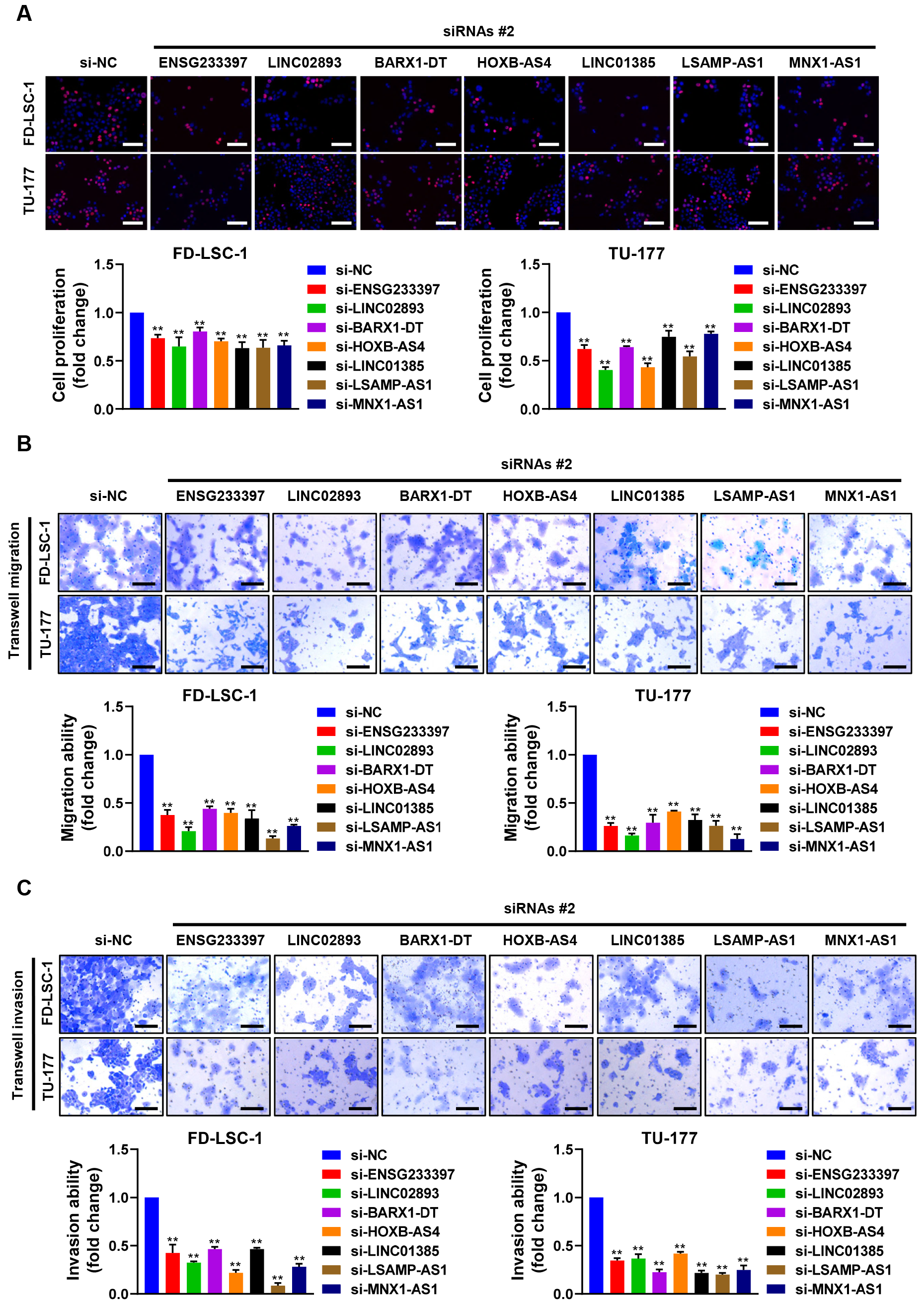

Supplement: Supplementary file 15 [file Image_10.tif]
